# The Role of Anethole in Reproductive Physiology and In Vitro Biotechnologies—A Review

**DOI:** 10.1111/rda.70176

**Published:** 2026-01-13

**Authors:** André Luiz da Conceição‐Santos, José Ricardo de Figueiredo

**Affiliations:** ^1^ Laboratory of Manipulation of Oocytes and Preantral Follicles, Faculty of Veterinary State University of Ceará Fortaleza Ceará Brazil

**Keywords:** Anethole, in vitro culture, reproductive biotechnologies, reproductive physiology

## Abstract

In vitro reproductive biotechnologies show promise for fertility preservation but still face challenges, including oxidative stress from high oxygen tension, which impairs cell viability and development. Antioxidants have been widely explored to attenuate oxidative stress during culture. Among them, anethole, a plant‐derived phenylpropanoid, stands out for its promising properties. This review explores the mechanisms and applications of anethole in reproductive physiology and its potential to enhance in vitro reproductive systems. Findings indicate that anethole modulates key pathways and may improve outcomes in in vitro follicle culture, oocyte in vitro maturation and in vitro embryo culture. These insights support future research and the strategic inclusion of anethole in reproductive biotechnology protocols.

## 1 Introduction

In vitro reproductive biotechnologies—including follicle culture (IVFC), oocyte maturation (IVM) and embryo culture (IVEC)—are hindered by variability in outcomes across species, driven in part by oxidative stress and suboptimal redox homeostasis. A central challenge in these systems is maintaining an appropriate redox balance, as elevated oxygen tension in vitro can induce oxidative stress, leading to increased cellular degeneration and compromised developmental competence. Consequently, antioxidant supplementation has become a widely employed strategy to mitigate the detrimental effects of reactive oxygen species (ROS) during in vitro culture (IVC) (Keane and Ealy 2024; Silva et al. 2023).

Among the array of compounds studied, anethole—a plant‐derived phenylpropanoid—has emerged as a particularly promising agent due to its diverse pharmacological properties, including anti‐inflammatory, antioxidant and cytoprotective effects (Sá et al. 2017). Nonetheless, despite substantial evidence from in vitro and in vivo studies in pharmacology and toxicology, few investigations have assessed the compound in the context of assisted reproduction (Conceição‐Santos, Velarde, et al. 2025). The precise mechanisms by which anethole modulates reproductive physiology remain incompletely understood and its targeted applications within in vitro reproductive systems are still largely unexplored.

This review aims to critically examine the current evidence on anethole's biological actions relevant to reproductive physiology, with a focus on its potential roles in IVFC, IVM and IVEC. By integrating findings from molecular, cellular and applied studies, we seek to provide a comprehensive framework to inform future experimental designs and the potential incorporation of anethole into reproductive biotechnology protocols. Through this synthesis, we aim to highlight both established knowledge and emerging opportunities, fostering innovative approaches that may advance reproductive outcomes in domestic and laboratory animals.

## 2 Physicochemical Characteristics of Anethole

Anethole is a lipophilic and volatile phenylpropanoid (C_10_H_12_O) that exists as two geometric isomers: cis‐(Z) and trans‐(E). The trans isomer is thermodynamically more stable and, therefore, the dominant form in natural sources (Marinov and Valcheva‐Kuzmanova 2015). Its physicochemical properties, such as low molecular weight (148.2 g/mol) and polarity, associated with high lipophilicity, enable efficient diffusion across biological membranes, thereby facilitating interactions with hydrophobic domains of proteins and lipid bilayers (Duță et al. 2019; Seo et al. 2024). These features are directly relevant to its pharmacological activity, as membrane integration and protein binding are critical determinants of bioactivity.

The compound exhibits a low melting point (20°C–21°C), rendering it liquid at physiological temperature and has a relatively high boiling point (231°C–237°C) (Clark 1993), which influences its stability in thermal processes. Its solubility is limited in aqueous media but high in organic solvents, which has important implications for experimental design and for the development of delivery systems intended to maximise its biological effects (Carteau et al. 2008; Raposo et al. 2024).

These physicochemical characteristics not only define its stability and reactivity but also shape its potential applications in reproductive biotechnology. For instance, its high lipophilicity may affect distribution within culture media, bioavailability to cells and its eventual incorporation into oocytes and embryos. As discussed below, these structural features underpin a broad spectrum of pharmacological activities and help explain its multiple biological effects.

## 3 Pharmacological Potential of Anethole

Owing to its unique physicochemical profile, anethole exerts a wide range of pharmacological activities, including anti‐inflammatory (Cao et al. 2022; Domiciano et al. 2013; Ghasemi‐dehnoo et al. 2022; Huang et al. 2024; Kang et al. 2013; Kim et al. 2017; Matboli et al. 2022; Moradi et al. 2014; Ponte et al. 2025; Ritter et al. 2013, 2017; Tong et al. 2022, 2023; Yu, Wang, Li, et al. 2022), neuroprotective (Khodadadian and Balali‐ Dehkordi 2025) and anticancer (Raposo et al. 2024). In the context of reproductive physiology and in vitro biotechnologies, however, two properties—its steroidogenic (Asadian et al. 2022; Chandran et al. 2019; Sá et al. 2020; Luo et al. 2020; Negahdari et al. 2021; Yancu et al. 2019) and antioxidant activities (Ghasemi‐dehnoo et al. 2022; Khoshnam et al. 2025; Mohamed et al. 2022; Noreen et al. 2023; Pandit et al. 2022; Rostami‐Faradonbeh et al. 2024; Ryu et al. 2014; Sá et al. 2019, 2020; Salimian et al. 2022; Vastegani et al. 2023; Yu, Wang, and Yang 2022)**—**are particularly relevant. Redox homeostasis and endocrine regulation are critical determinants of follicular development, gamete competence and embryonic viability, making these mechanisms highly significant for reproductive outcomes.

## 4 Antioxidant Properties and Implications for Reproductive Biotechnology

As summarised in Table 1, anethole demonstrates potent antioxidant effects across diverse in vivo and in vitro models. Notably, it reduces malondialdehyde (MDA) levels—a biomarker of lipid peroxidation—in reproductive tissues and systemic compartments, suggesting a protective role against oxidative damage to oocytes and follicular cells. Concurrently, anethole enhances ferric reducing antioxidant power (FRAP) in cultured early antral follicles and their microenvironment, indicating a capacity to bolster intrinsic antioxidant defences during oocyte maturation. These observations are particularly relevant for in vitro embryo production systems, where oxidative stress is a well‐documented impediment to blastocyst yield and quality (Keane and Ealy 2024; Rakha et al. 2022).

**TABLE 1:** Effects of anethole on antioxidant markers in multiple in vivo and in vitro models.

Antioxidant marker	Effect of anethole	Main results/potential impacts on follicles, oocytes and embryos	References
MDA	↓ MDA in multiple tissues^a^	↓ MDA/Reduces lipid peroxidation, thereby protecting oocyte membranes, follicular cells and embryonic viability	Al‐Ali et al. (2024), Ghasemi‐dehnoo et al. (2022), Mohamed et al. (2022), Negahdari et al. (2022), Noreen et al. (2023), Pandit et al. (2022), Rostami‐Faradonbeh et al. (2024), Salimian et al. (2022), Samadi‐Noshahr et al. (2021), Vastegani et al. (2023), Younis and Mohamed (2023), Yu, Wang, and Yang (2022)
FRAP	↑ FRAP in tissues^b^ and cells^c^	↑ FRAP/Enhances antioxidant capacity, thereby supporting oocyte maturation, follicular growth and embryonic development	Ghasemi‐dehnoo et al. (2022), Rostami‐Faradonbeh et al. (2024), Sá et al. (2020), Sá et al. (2019), Salimian et al. (2022)

*Note:* Data from multiple in vivo and in vitro models indicate anethole's antioxidant potential that can be translated to follicles, oocytes and embryo culture conditions.

Abbreviations: FRAP, Ferric Reducing Antioxidant Power; MDA, Malondialdehyde.

^a^ Tissues with reduced MDA: cardiac, renal, colon, ovarian, blood, liver, hippocampus, prefrontal cortex, brain striatum, jejunum and ileum.

^b^ Tissues with increased FRAP: colon, hippocampus and prefrontal cortex.

^c^ Cells with increased FRAP: COC and early antral follicles (detected in the medium after in vitro culture).

## 5 Steroidogenic Modulation

Anethole's influence on steroidogenesis is complex and depends on the biological context, as shown data in Table 2. The key findings can be summarised as follows:

- Estrogenic activation: In ovarian follicles, anethole boosts the activity of the Cyp19A1 (aromatase) enzyme, increasing estradiol (E2) production. This may support follicular growth in species where oestrogen is crucial for this process, such as domestic mammals.
- Anti‐androgenic effects: In models of Polycystic Ovary Syndrome (PCOS), anethole significantly reduced levels of hormones like testosterone (T) and dehydroepiandrosterone (DHEA). This highlights its potential for managing similar endocrine disorders in domestic animals.
- Broad modulation of hormones: In H295R and BeWo cell models, anethole increased the expression of key genes involved in steroid biosynthesis, including StAR and Cyp11A1. This led to increased synthesis of multiple hormones, including progesterone (P4) androstenedione (A4, an androgen precursor) and oestrogens (E1, E2, E3). This indicates that anethole's effects are not uniform and depend on the specific cell type or species, warranting further investigation on such effects.

**TABLE 2:** Effects of anethole on steroidogenesis and reproductive hormones.

Model system	Species	Biological material	Key outcomes	References
In vivo	Murine	Ovary	↑ *Cyp19A1* gene expression → potential support for oestrogen‐dependent follicular development.	Asadian et al. (2022)
In vitro	Human	Sperm	↓ Sperm activation → possible implications for sperm function regulation.	Luo et al. (2020)
In vivo (PCOS model)	Murine	Blood	↓ T and DHEA → potential therapeutic role in PCOS.	Negahdari et al. (2021)
In vitro	Caprine	Early antral follicle	↑ *Cyp19A1, Cyp17* gene expression and E2 levels in culture medium → Promotes follicular oestrogen synthesis, which may improve oocyte maturation.	Sá et al. (2020)
In vitro	Human	H295R/BeWo cells	↑ E1, E2, E3, P4, DHEA, A4 in the co‐culture medium and *StAR, Cyp3A7* and *Cyp19 P2* gene expressions in H295R cells → Broad steroidogenic modulation suggests multi‐target action in hormone‐producing cells.	Yancu et al. (2019)

Abbreviations: A4, androstenedione; BeWo, human trophoblastic choriocarcinoma cell line; Cyp19A1, aromatase; Cyp19 P2, promoter 2 of the Cyp19 gene; Cyp3A7, cytochrome P450 3A7; DHEA, dehydroepiandrosterone; E1, estrone; E2, estradiol; E3, estriol; H295R, human adrenocortical carcinoma cell line; P4, progesterone; PCOS, polycystic ovary syndrome; StAR, steroidogenic acute regulatory protein; T, testosterone.

Despite strong evidence from in vitro and in vivo studies, most available data on anethole's steroidogenic modulation remain fragmented across different biological systems. In this perspective, a significant challenge is finding ways to directly improve outcomes for oocytes and embryos in a lab setting. Closing this gap requires determining the right dose and delivery method for anethole in reproductive culture systems.

## 6 Estrogenic Activity

Oestrogen plays a central role in female reproductive physiology and, together with progesterone, constitutes one of the primary sex hormones in females. Endogenously, oestrogens are synthesised from cholesterol through steroidogenic pathways. However, certain exogenous compounds can also exhibit oestrogen‐like activity due to their chemical properties and structural similarity to endogenous oestrogens. Such compounds are classified as phytoestrogens. Beyond its regulatory role in steroidogenesis, anethole has been described as a phytoestrogen.

Anethole shows demonstrable but moderate interaction with oestrogen receptors (ER). For example, in the Yeast Oestrogen Screen—a bioassay in which genetically modified yeast express the human oestrogen receptor—it acts as a partial agonist, reaching about half of the maximal estrogenic response (54.5%) when used at relatively high concentration (EC₅₀ = 625 μg/mL) compared to 17β‐estradiol (Tabanca et al. 2004). Although plant extracts containing anethole have shown to reproduce these estrogenic effects (Albert‐Puleo 1980), the specific contribution of anethole itself still requires confirmation through studies using purified compound and mammalian ER isoforms (ERα/ERβ). In addition, the precise binding model of anethole and its metabolites remains insufficiently characterised and warrants targeted ligand‐binding studies.

The sex‐divergent effects reported for anethole are particularly noteworthy: In females, its estrogenic potential may complement anethole's steroidogenic regulation; however, direct evidence linking ER activation to specific ovarian outcomes remains limited. In males, by contrast, studies report dose‐dependent disruptions of reproductive function triggered by anethole exposure, including:

- Reduced sperm count and suppressed gonadotropins (FSH, LH) in rats (Helal et al. 2019);
- Inhibition of sperm capacitation through impaired Ca^2+^ signalling and tyrosine phosphorylation (Luo et al. 2020).

These contrasting effects occur primarily at pharmacological concentrations, leaving open questions about the threshold between therapeutic and adverse effects, ER subtype selectivity (α vs. β) in different tissues and the relevance of these findings to clinical or biotechnological applications.

## 7 Natural Sources of Anethole

Anethole occurs naturally in various plant species, predominantly in the trans‐(E) isomeric form (Marinov and Valcheva‐Kuzmanova 2015). Interestingly, its name derives from *Anethum graveolens* L., a plant that paradoxically contains only trace amounts of the compound (Duță et al. 2019). The main sources of anethole are the essential oils from star anise ( *Illicium verum* Hook. f.), whose oil contains 75.5%–99.9% of the compound (Huang et al. 2010; Lei et al. 2023; Yang et al. 2021); anise ( *Pimpinella anisum* L.), whose oil contains 74.8%–99.2% (Duță et al. 2019; Skalicka‐Woźniak et al. 2013; Zeni et al. 2025); and fennel ( *Foeniculum vulgare* Mill.), whose oil contains 30.7%–90.4% (Duță et al. 2019; Senatore et al. 2013). In Brazil, species of the genus *Croton*, such as *C. zehntneri* Pax & K. Hoffm. (‘canela de cunhã’) and *C. grewioides* Müll. Arg. (‘canelinha de cheiro’), have also been identified as rich sources, accounting for 85.7% (Oliveira et al. 2001) and 96.7% (Silva et al. 2024) of their respective essential oils. Other notable sources include *Backhousia anisata* vickery., endemic to Australia (Blewitt and Southwell 2000) and Asian species of *Clausena*, such as *C. harmandiana* Pierre (Peerakam et al. 2022) and *C. heptaphylla* (Roxb.) Wight & Arn. (Lal et al. 2022).

However, concentrations of anethole vary considerably among and within species, influenced by factors such as the plant part used for extraction, soil composition, climate and geographical origin (Blewitt and Southwell 2000; Duță et al. 2019; Senatore et al. 2013). Additional factors, including the type of extraction technique employed and the analytical method utilised, can also contribute to the final composition of anethole isolated. Among the studies referenced, hydrodistillation and solvent extraction were commonly employed, while quantification typically relies on gas chromatography (with or without mass spectrometry) or proton nuclear magnetic resonance.

This variability presents a critical challenge for standardisation in reproductive biotechnology, where reproducible dosing is essential to ensure predictable effects on oocytes and embryos. For instance, in a recent study using essential oil extracted from *C. grewioides* Müll. Arg. supplementation at 1 μg/mL promoted the development of morphologically normal follicles and increased collagen fibre area in bovine ovarian tissue cultures. Conversely, when using pure synthetic anethole, similar effects on collagen fibres were observed only at 1000 μg/mL (Silva et al. 2024). This striking difference illustrates how the composition and concentration of natural versus synthetic sources can dramatically alter outcomes in culture systems.

While the diversity of natural sources underscores the accessibility of anethole, further studies are needed to establish reliable extraction and quantification protocols tailored for reproductive applications. Such standardisation is crucial for bridging the gap between pharmacological evidence and experimental reproducibility in assisted reproduction.

## 8 Anethole‐Modulated Signalling Pathways Relevant to Reproductive Physiology

**FIGURE 1:**
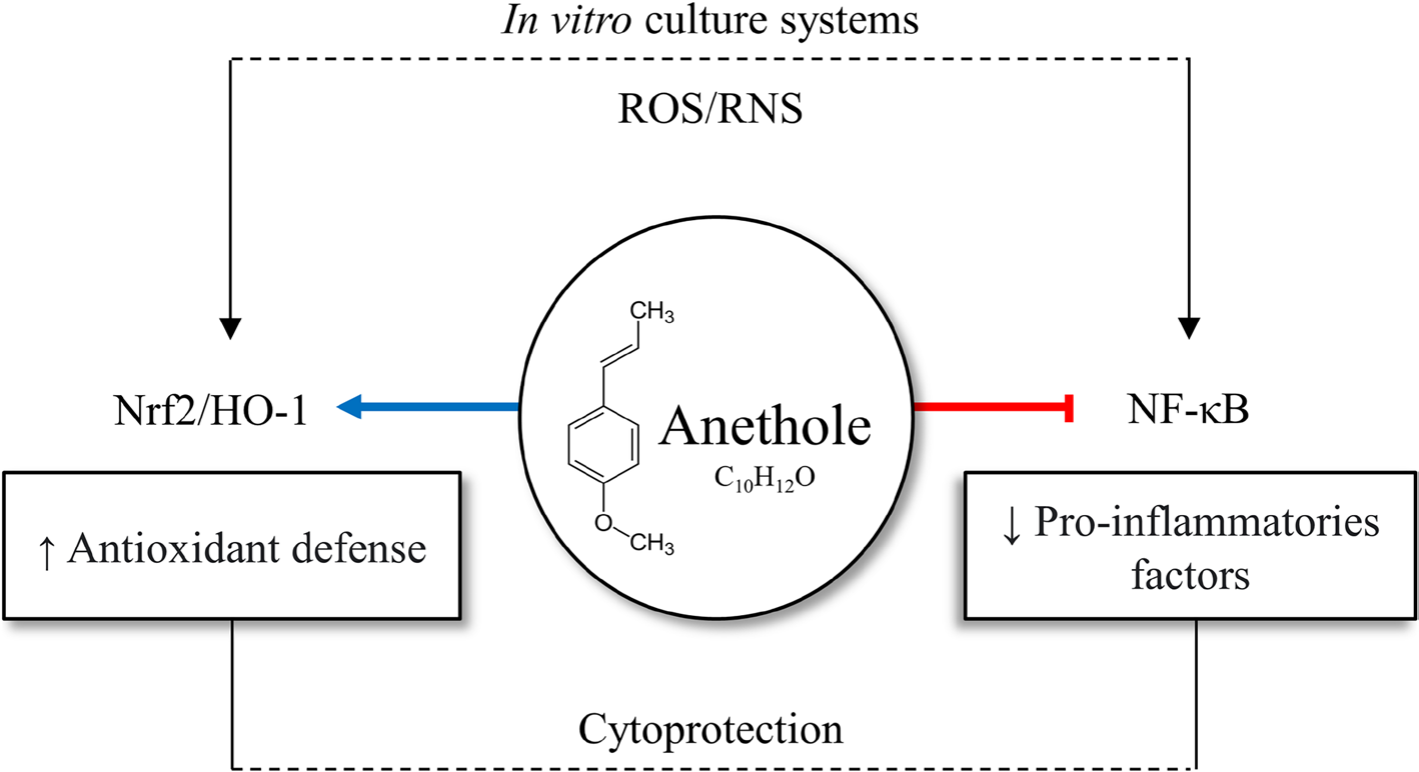
Schematic representation of the main biological effects of anethole (C_10_H_12_O). Anethole enhances antioxidant defences and reduces oxidative stress, while also downregulating pro‐inflammatory markers and promoting cytoprotection. These dual actions underline its potential role in modulating oxidative and inflammatory pathways. Blue arrow indicates stimulatory effect, whereas red arrow that end with a line indicates inhibitory effect. ROS, Reactive oxygen species; RNS, Reactive nitrogen species.

Beyond its pharmacological and physicochemical properties, anethole interacts with multiple cellular signalling pathways that are intimately linked to reproductive physiology. By modulating these pathways, anethole may influence key processes such as follicular activation, oocyte maturation, embryonic development and cellular redox homeostasis, underscoring its potential utility in reproductive biotechnologies. Figure 1 shows the main biological effects of anethole through the pathways in which its actions have been most explored, highlighting its antioxidant and anti‐inflammatory properties. Beyond these pathways, anethole has also been reported to modulate several signalling pathways relevant to cell development and survival, as shown below.

### 8.1 PI3K/Akt/mTOR Pathway

The PI3K/Akt/mTOR signalling axis is a highly conserved pathway that regulates key cellular processes such as growth, metabolism, differentiation, proliferation and survival in response to various external and internal stimuli. PI3K acts by activating Akt and Akt subsequently activates the mammalian target of rapamycin (mTOR), which acts as a major downstream controller of cell activity (He et al. 2021). mTOR functions operate within two distinct multiprotein complexes—mTOR complex 1 (mTORC1) and mTOR complex 2 (mTORC2)—each with specific regulatory roles (Simcox and Lamming 2022). mTORC1 primarily regulates cell growth and metabolism, whereas mTORC2 contributes to cell survival and proliferation.

In reproductive physiology, activation of the PI3K/Akt pathway and its downstream effector mTOR is essential for several fundamental events, including: recruitment and growth of ovarian follicles, maintenance of meiotic arrest prior to the luteinizing hormone (LH) surge, meiotic resumption and early embryonic development (Guo et al. 2018; Guo and Yu 2019; Han et al. 2023; Liu et al. 2024; Liu et al. 2022; Su et al. 2022; Sun et al. 2015; Zhang et al. 2022).

Anethole has been described as a modulator of PI3K/Akt/mTOR signalling in diverse cellular contexts. For instance, Rhee et al. (2018) showed that anethole inhibited adipogenic differentiation of human mesenchymal stem cells by suppressing the Akt–mTOR‐p70S6K‐PPARγ axis under oxidative stress, illustrating its capacity to influence stress‐responsive pathways. Similarly, Yadollahi‐Farsani et al. (2024) reported that intraperitoneal administration of anethole upregulated PI3K, Akt and mTOR gene expression in the hippocampus of maternally separated mice, improving behavioural outcomes. Although these findings confirm that anethole can modulate PI3K/Akt/mTOR signalling in non‐reproductive tissues, its direct role in folliculogenesis, oocyte maturation and early embryonic development remains unknown, underscoring the need for reproductive‐specific studies.

### 8.2 NF‐κB Pathway

Nuclear factor kappa B (NF‐κB) is a pivotal transcription factor that regulates more than 400 genes involved in immune responses, inflammation, cell proliferation and survival. Under resting conditions, NF‐κB remains inactive in the cytoplasm—including within the oocyte ooplasm—because it is bound to inhibitory IκB proteins. When cells encounter activating stimuli such as lipopolysaccharides (LPS) or oxidative stress, IκB proteins are phosphorylated and degraded, releasing NF‐κB. This allows NF‐κB to move into the nucleus and activate its target genes (Feng et al. 2019).

In female reproduction, NF‐κB plays a dual role. On the one hand, it contributes to physiological processes such as ovulation and parturition by upregulating inflammatory mediators, including cyclooxygenase‐2 (COX‐2) and matrix metalloproteinases (MMPs), which facilitate follicular rupture and uterine contractions (Guan et al. 2021; Gómez‐Chávez et al. 2021). On the other hand, excessive NF‐κB activation under conditions of oxidative stress—commonly found during in vitro culture—may elevate proinflammatory cytokine production, compromise follicle and oocyte quality and reduce embryo competence. This imbalance may amplify oxidative damage and increase cellular injury (Wang et al. 2020; Wright et al. 2020; Xu et al. 2024).

In this context, anethole has shown consistent anti‐inflammatory and cytoprotective activity through inhibition of NF‐κB signalling. Evidence from non‐reproductive models shows that anethole can suppress NF‐κB activation by inhibiting the TLR4/NF‐κB pathway in IEC‐6 cells exposed to LPS (Tong et al. 2022), preventing IκB degradation in jejunal inflammation of broilers (Tong et al. 2023) and reducing TNF‐α by blocking IκB‐α degradation (Kang et al. 2013). Additional studies show similar inhibitory effects on NF‐κB and MAPK pathways in cancer and colitis models (Choo et al. 2011; Contant et al. 2021), as well as protection against TNF‐induced apoptosis through suppression of NF‐κB activation (Chainy et al. 2000).

Taken together, these findings suggest that NF‐κB is a key mechanistic target through which anethole may exert beneficial effects in reproductive systems. Specifically, in assisted reproduction—particularly in vitro follicle and embryo culture, where inflammatory and oxidative stressors are heightened—anethole's ability to modulate NF‐κB could help preserve follicular integrity, improve oocyte competence and enhance embryo developmental outcomes. Nonetheless, direct experimental evidence in reproductive studies is still required to confirm these proposed benefits.

### 8.3 Nrf2/HO‐1

The nuclear factor erythroid 2–related factor 2 (Nrf2) is a central transcription factor that coordinates cellular defences against oxidative stress by regulating redox balance, antioxidant responses and detoxification pathways (Loboda et al. 2016). Reactive oxygen and nitrogen species (ROS/RNS) are generated in multiple cellular compartments, either as normal by‐products of aerobic metabolism—primarily in mitochondria—or in response to toxic or pathological challenges (Ma 2013). Although *Nrf2* mRNA is constitutively expressed, its activity is tightly controlled at the post‐transcriptional level. Increases in ROS and other stimuli induce conformational changes in Kelch‐like ECH‐associated protein 1 (Keap1), stabilising Nrf2 and enabling its translocation to the nucleus, where it activates target genes (O'Rourke et al. 2024).

Once activated, Nrf2 binds to the antioxidant response element (ARE, 5′‐TGACnnnGC‐3′), thereby inducing the transcription of cytoprotective genes such as heme oxygenase‐1 (HO‐1) (Ngo and Duennwald 2022). HO‐1 catalyses the degradation of heme, an essential component of mitochondrial electron transport, into biliverdin, carbon monoxide and free iron. By promoting ferritin synthesis and iron detoxification, HO‐1 prevents Fenton‐driven radical formation and protects cellular integrity (Campbell et al. 2021; Waza et al. 2018). The Nrf2/HO‐1 axis has therefore emerged as a central pathway in reproductive physiology and pathology, since redox imbalance critically influences gamete competence, follicular development and embryo viability.

Anethole has been reported to modulate the Nrf2/HO‐1 pathway in diverse biological contexts. In reproductive models, supplementation with anethole or anethole‐rich essential oils during IVC of bovine preantral follicles enhanced catalase activity, reducing oxidative stress, but paradoxically downregulated Nrf2 mRNA expression (Silva et al. 2024). This suggests that under low oxidative stress conditions, Nrf2 is attenuated, highlighting the complexity of its regulatory dynamics in assisted reproduction. Beyond the reproductive field, in vivo studies have shown that anethole promotes cardioprotection by upregulating Nrf2 and HO‐1 protein expression in a mouse model of myocardial infarction, leading to reduced oxidative stress, inflammation and apoptosis (Younis and Mohamed 2022). Similarly, in poultry models of necrotic enteritis, anethole enhanced intestinal integrity through Nrf2/HO‐1 upregulation and concomitant increases in SOD1 and GSH levels (Yu, Tong, Li, et al. 2022).

Taken together, these findings indicate that anethole may act as a conditional regulator of the Nrf2/HO‐1 pathway, with its effects depending on baseline oxidative status and tissue context. In reproductive systems—particularly in assisted reproduction and in vitro culture conditions, where oxidative imbalance is a major limiting factor—the capacity of anethole to modulate Nrf2/HO‐1 could represent a valuable mechanism to preserve follicular and oocyte quality, as well as to improve embryo developmental potential. Nonetheless, further targeted studies are necessary to validate these effects.

### 8.4 MAPK/ERK

Mitogen‐Activated Protein Kinase (MAPK) refers to a family of kinase cascades—including Extracellular Signal‐Regulated Kinase (ERK)—involved in key cellular processes such as proliferation, differentiation and survival. These signalling pathways are particularly relevant in reproductive biology, since they are active in oocytes, follicular cells and embryos (Liang et al. 2007; Lu et al. 2008).

Although the regulation of MAPK family members by anethole has not yet been assessed in the context of reproductive physiology or biotechnologies, some experimental models provide relevant insights. For instance, Qu et al. (2021) showed that anethole inhibited ERK phosphorylation in a dose‐ and time‐dependent manner, thereby suppressing osteoclast differentiation induced by RANKL and preventing bone loss in ovariectomized mice—a well‐established model of oestrogen deficiency. Interestingly, this effect was specific to ERK, since p38 and JNK pathways were not altered in the same context. In addition, anethole exerted protective effects against hepatic ischemia in mice by modulating ERK, p38 and JNK (Cho et al. 2013), suggesting a context‐dependent selectivity in MAPK regulation.

The role of anethole in MAPK signalling has also been investigated in cancer models, where activation of ERK, p38 and JNK—MAPK family members—is tightly associated with uncontrolled proliferation and cell survival. In oral cancer cell lines, anethole suppressed ERK1/2, p38 and JNK phosphorylation, displaying strong antitumor activity (Contant et al. 2021). Similarly, in human fibrosarcoma cells, anethole acted as an anti‐metastatic agent by downregulating ERK and p38 during invasion assays (Choo et al. 2011).

Taken together, these findings demonstrate that anethole can regulate multiple MAPK/ERK‐related signalling nodes, although its effects appear to be highly context‐ and cell‐type dependent. This variability highlights both the compound's pleiotropic potential and the challenges in extrapolating non‐reproductive data to gametes and embryos. In particular, the absence of direct studies on oocytes, cumulus cells, or embryos prevents definitive conclusions about how MAPK/ERK modulation by anethole could influence key reproductive events such as meiotic progression, cytoplasmic maturation, or early embryonic development. Addressing these gaps represents a critical step toward clarifying the translational relevance of anethole in reproductive biotechnologies.

## 9 Implications of Anethole in In Vitro Reproductive Biotechnologies

Although the estrogenic and steroidogenesis‐regulating properties of anethole were described prior to the 1980s (Albert‐Puleo 1980) and its antioxidant effects have been widely investigated in different biological systems, it was only in the last decade that its role in in vitro reproductive biotechnologies—including IVFC, IVM of oocytes and IVEC—began to be systematically evaluated.

**FIGURE 2:**
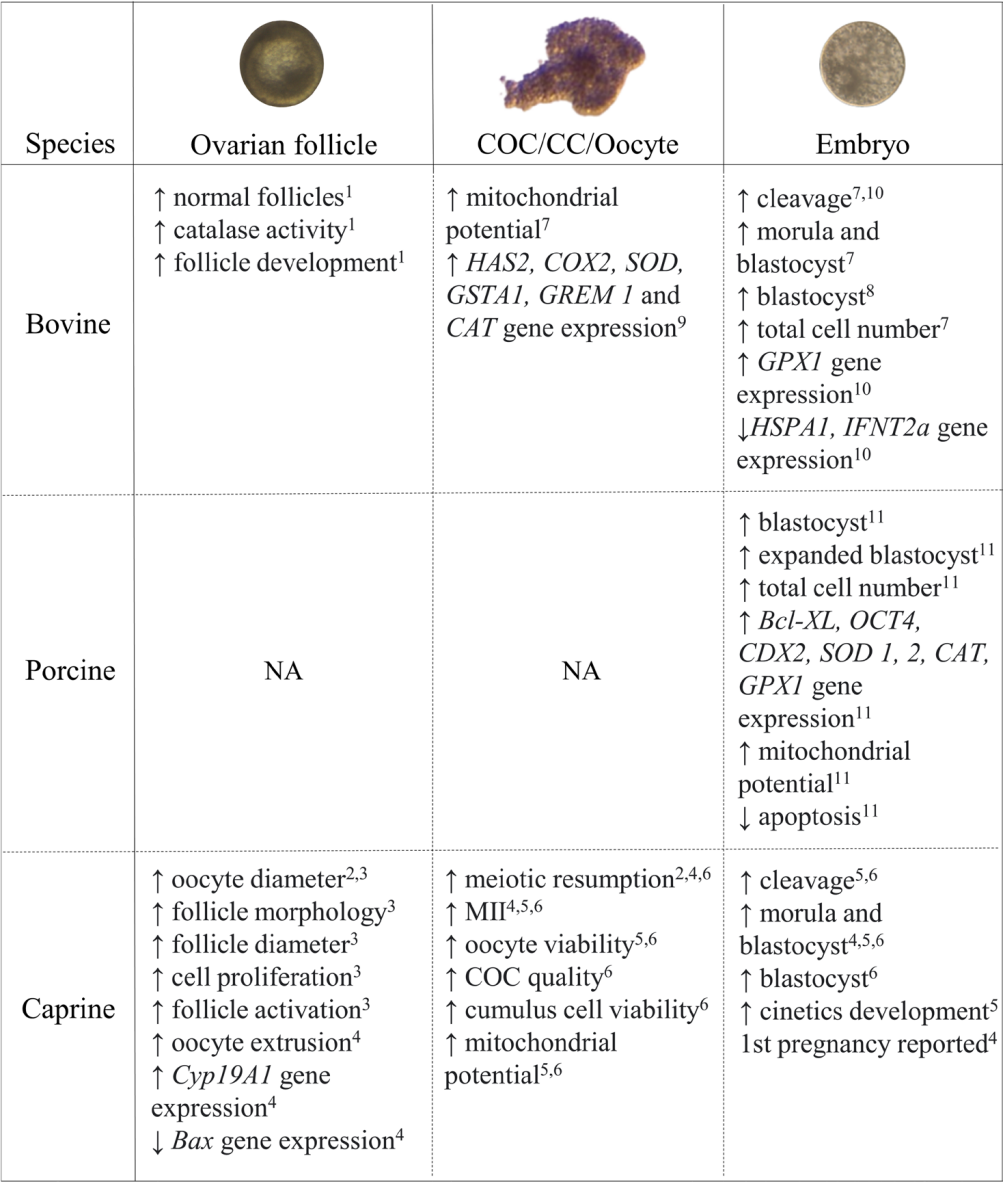
Main findings obtained with anethole supplementation in in vitro reproductive biotechnologies. Superscript indicate the effects of anethole following different culture systems designed for ^1^bovine ovarian tissue; ^2^isolated caprine secondary follicles; ^3^caprine ovarian tissue; ^4^isolated caprine early antral follicles; ^5^caprine COCs; ^6^early antral follicles and caprine COCs; ^7^bovine COCs; ^8^bovine embryos; ^9^bovine COCs; ^10^bovine COCs and/or embryos; ^11^porcine embryos. BAX, BCL2‐associated X protein (pro‐apoptotic protein); BCL‐XL, B‐cell lymphoma‐extra‐large (anti‐apoptotic protein); CAT, catalase; CC, cumulus cell; CDX2, caudal type homeobox 2 (trophectoderm marker); COC, cumulus–oocyte complex; COX2, cyclooxygenase 2; GPX1, glutathione peroxidase 1; GREM1, gremlin 1 (BMP antagonist); GSTA1, glutathione S‐transferase alpha 1; HAS2, hyaluronan synthase 2; MII, metaphase II oocyte; NA, not applicable; OCT4, octamer‐binding transcription factor 4 (pluripotency marker); SOD, superoxide dismutase.

The initial rationale for introducing anethole into these protocols was its antioxidant potential (Sá et al. 2017), in response to one of the major limitations of assisted reproductive technologies in vitro: the difficulty in maintaining redox homeostasis. Imbalances in the oxidising–reducing environment are known to compromise gamete and embryo development (Keane and Ealy 2024). Against this backdrop, anethole was proposed as a culture medium supplement capable of improving reproductive outcomes by mitigating oxidative stress. As summarised in Figure 2, anethole supplementation has been associated with multiple effects on oocyte competence, embryo development and molecular markers in in vitro reproductive biotechnologies. Interestingly, although not universally, several outcomes influenced by anethole in major animal‐reproduction studies were concentration‐dependent. Overall, the concentrations tested ranged from 1 to 3000 μg/mL and the effects varied according to both species and dose, as recently reported (Conceição‐Santos, Velarde, et al. 2025; Conceição‐Santos, Silva, et al. 2025). A more detailed discussion of anethole's contribution to reproductive biotechnologies is presented below.

### 9.1 Anethole and In Vitro Follicle Culture

Pioneering studies by Sá et al. (2017) showed that anethole supplementation during IVFC of goat isolated secondary follicles modulated key developmental parameters. At 30 μg/mL, it increased the proportion of fully grown oocytes, while at 2000 μg/mL, it enhanced meiotic resumption. Both concentrations reduced ROS levels in the culture medium compared with ascorbic acid. In goat ovarian tissue culture, anethole improved follicular morphology, oocyte and follicle diameter and promoted proliferation (via PCNA upregulation), suggesting an effect not only on oxidative status but also on follicular activation and growth (Sá et al. 2018). In bovine ovarian tissue culture, both anethole and *C. grewioides* essential oil increased the percentage of morphologically normal follicles and collagen fibres. At 1 μg/mL, *C. grewioides* essential oil increased the number of stromal cells, glutathione peroxidase activity and thiol levels, whereas the same concentration of anethole increased catalase activity and reduced glutathione peroxidase activity (Silva et al. 2024), suggesting distinct mechanisms by which anethole and anethole‐rich essential oils modulate ROS in ovarian tissues.

A critical milestone came with the report of the first pregnancy achieved after transfer of an embryo produced entirely in vitro (by IVFC, oocyte IVM and IVEC) in caprine species with anethole supplementation (Sá et al. 2020). In this context, anethole improved redox balance (FRAP), increased estradiol secretion, upregulated steroidogenic genes (*Cyp17, Cyp19A1*) and reduced pro‐apoptotic signalling (*Bax/Bcl‐2* ratio), indicating combined antioxidant, steroidogenic and cytoprotective effects.

A recent study demonstrated that supplementing anethole during both IVFC and IVM significantly enhances oocyte quality, cumulus‐oocyte complex integrity, cumulus cell viability, antioxidant capacity and mitochondrial activity. These improvements translated into higher oocyte maturation rates and embryo production yields, closely mirroring in vivo outcomes in goat species (Conceição‐Santos, Silva, et al. 2025). Such findings highlight anethole's potential as a strategic supplement to overcome a critical limitation of follicular culture—the inability to consistently generate oocytes competent for full‐term embryonic development. However, further research is needed to validate its efficacy across different species and culture systems, as well as to elucidate the underlying molecular mechanisms.

### 9.2 Anethole and Oocyte In Vitro Maturation

In bovine oocytes, 300 μg/mL of anethole increased mitochondrial membrane potential, cleavage and blastocyst rates after in vitro fertilisation and FRAP in the culture medium after IVM (Sá et al. 2019). Gene expression analyses revealed upregulation of oxidative stress resistance genes (*GSTA1, SOD1, CAT*) and cumulus expansion markers (*HAS2, COX2*) at 300–3000 μg/mL in bovine cumulus cells after oocyte IVM (Janini et al. 2023). However, the highest concentration impaired oocyte maturation, emphasising the importance of dose–response relationships.

Similarly, in caprine oocytes, 30 μg/mL improved maturation, cleavage and embryo development, while 2000 μg/mL had detrimental effects. Interestingly, mitochondrial activation at 2000 μg/mL occurred without changes in ROS, suggesting possible metabolic overstimulation (Conceição‐Santos et al. 2024). Collectively, these findings highlight the dual potential of anethole—beneficial at moderate doses, but harmful at higher concentrations and underscores the need for species‐specific optimization.

### 9.3 Anethole and Embryo In Vitro Culture

During embryo IVC, anethole also demonstrated species‐specific benefits. In bovine embryos, 30 μg/mL increased blastocyst formation (Anjos et al. 2019). In porcine embryos, 500 μg/mL improved blastocyst quality, increased cell number, reduced apoptosis, enhanced mitochondrial activity and modulated lipid metabolism (Joo et al. 2023). Notably, this study was the first to link anethole supplementation with activation of the SHH pathway, a key regulator of embryogenesis (Carballo et al. 2018). This finding expands the mechanistic basis of anethole's action beyond redox regulation.

## 10 Conclusion and Perspectives

Anethole emerges as a promising bioactive compound for in vitro reproductive biotechnologies, particularly due to its antioxidant, anti‐inflammatory and steroidogenic properties. Evidence to date indicates that supplementation with anethole can improve oocyte quality, enhance in vitro maturation and support embryonic development, although these effects are strongly dependent on concentration, species and experimental conditions. Its dual dose‐dependent potential—beneficial at moderate levels but potentially detrimental at higher concentrations—underscores the need for careful optimization.

Despite these encouraging findings, the molecular mechanisms underlying anethole's actions remain incompletely elucidated and data on physiologically relevant doses, long‐term safety and reproducibility are still limited. Future studies should focus on standardising culture protocols, clarifying intracellular signalling targets and validating translational applications across domestic animal species.

**FIGURE 3:**
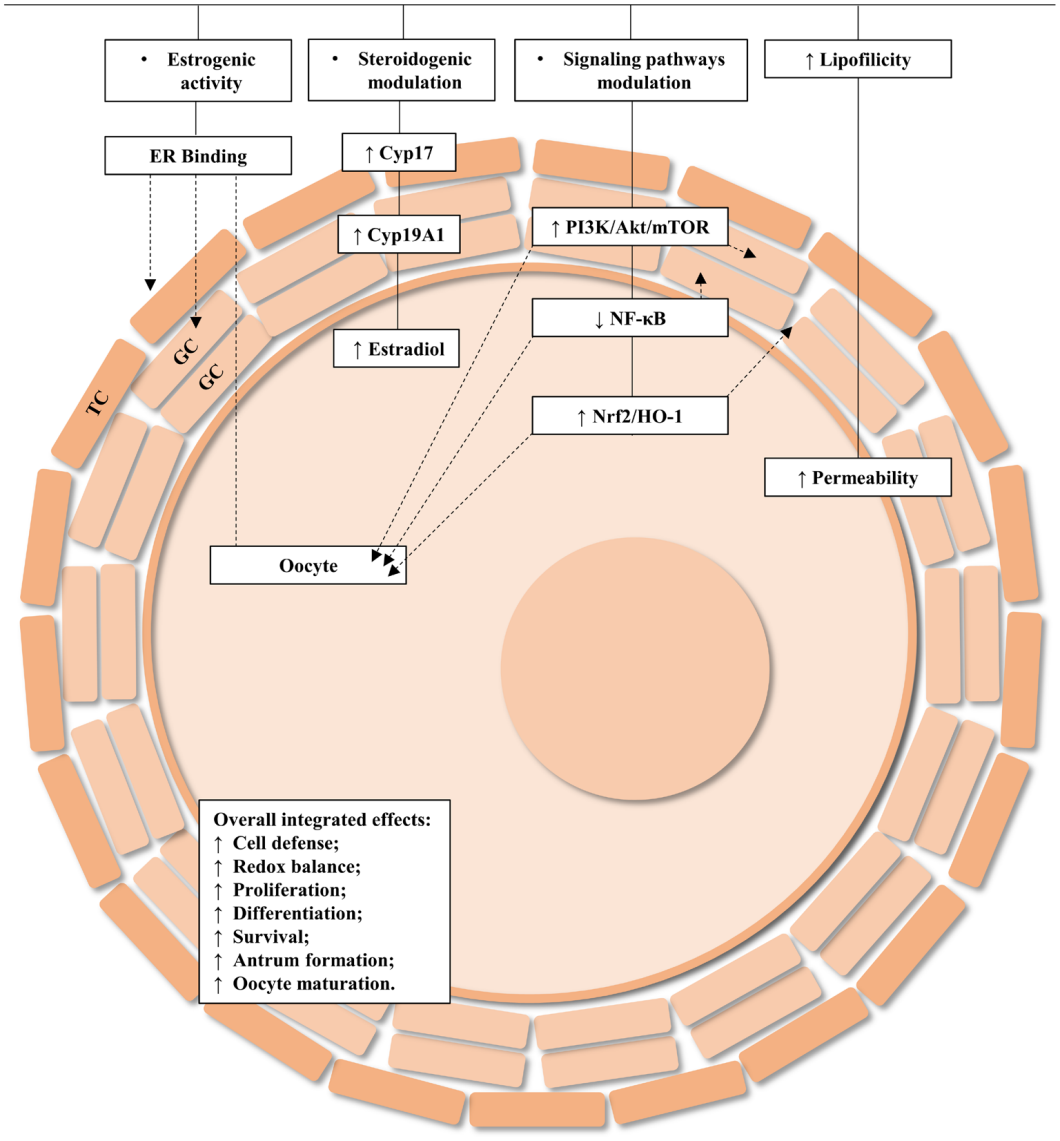
Proposed mechanistic actions of anethole in the follicular compartments and its overall integrated effects. Anethole's physicochemical properties and oestrogen receptor–binding capacity facilitate its entry into follicular cells, enabling modulation of inflammatory (NF‐κB), survival/proliferation (PI3K/AKT/mTOR) and antioxidant (Nrf2/HO‐1) signalling pathways. In theca cells, anethole may enhance steroidogenesis through upregulation of Cyp17, increasing androgen availability. In granulosa cells, anethole promotes aromatase (Cyp19A1) expression and estradiol synthesis, while supporting proliferation, differentiation and anti‐apoptotic activity. In the oocyte, anethole may contribute to improved cytoplasmic protection, metabolic cooperation and oocyte maturation. Together, these coordinated actions result in overall integrated effects, including enhanced cell defence, redox balance, follicular growth, antrum formation and developmental competence of the oocyte. GC, granulosa cell; TC, theca cell.

Overall, anethole represents a valuable candidate for incorporation into IVFC, IVM and IVEC protocols, with the potential to enhance reproductive outcomes. Further robust research is essential to ensure both efficacy and biosafety, ultimately bridging the gap between experimental evidence and practical application in reproductive biotechnology. A concise visual model summarising the proposed mechanistic actions of anethole across follicular compartments is presented in Figure 3 to assist readers in integrating the key concepts discussed throughout this review.

## Author Contributions

André Luiz da Conceição Santos conducted the literature review, designed the manuscript and wrote the paper. José Ricardo de Figueiredo supervised and reviewed the manuscript. Both authors have read and agreed to the published version of the manuscript.

## Conflicts of Interest

The authors declare no conflicts of interest.

## Data Availability

Data sharing not applicable to this article as no datasets were generated or analysed during the current study.

## References

[rda70176-bib-0001] Al‐Ali, M. A. , N. S. Younis , B. Aldhubiab , A. S. Alatawi , M. E. Mohamed , and M. S. Abd El Dayem . 2024. “Anethole Alleviates Doxorubicin‐Induced Cardiac and Renal Toxicities: Insights From Network Pharmacology and Animal Studies.” Chemico‐Biological Interactions 401: 11115. 10.1016/j.cbi.2024.111155. 39029857

[rda70176-bib-0002] Albert‐Puleo, M. 1980. “Fennel and Anise as Estrogenic Agents.” Journal of Ethnopharmacology 2, no. 4: 337–344. 10.1016/S0378-8741(80)81015-4. 6999244

[rda70176-bib-0003] Anjos, J. C. , F. L. N. Aguiar , N. A. R. Sá , et al. 2019. “Anethole Improves Blastocysts Rates Together With Antioxidant Capacity When Added During Bovine Embryo Culture Rather Than in the *in Vitro* Maturation Medium.” Zygote (Cambridge, England) 27, no. 6: 382–385. 10.1017/S0967199419000443. 31451120

[rda70176-bib-0004] Asadian, M. , H. Yaghoubi , F. Mahmoudi , and K. Haghighat Gollo . 2022. “Effect of Trans‐Anethole on Gene Expression of Steroidogenic Enzymes in the Ovary of Polycystic Ovary Syndrome Model Rate.” Galen Medical Journal 11. 10.31661/gmj.v11i.2219. PMC10188247 37200684

[rda70176-bib-0005] Blewitt, M. , and I. A. Southwell . 2000. “Backhousia Anisata Vickery, an Alternative Source of (E)‐Anethole.” Journal of Essential Oil Research 12, no. 4: 445–454. 10.1080/10412905.2000.9699563.

[rda70176-bib-0006] Campbell, N. K. , H. K. Fitzgerald , and A. Dunne . 2021. “Regulation of Inflammation by the Antioxidant Haem Oxygenase 1.” Nature Reviews Immunology 21, no. 7: 411–425. 10.1038/s41577-020-00491-x. 33514947

[rda70176-bib-0007] Cao, Y. , Q. Zhong , F. Tang , X. Yao , Z. Liu , and X. Zhang . 2022. “Anethole Ameliorates Inflammation Induced by Monosodium Urate in an Acute Gouty Arthritis Model via Inhibiting TLRs/MyD88 Pathway.” Allergologia et Immunopathologia 50, no. 6: 107–114. 10.15586/aei.v50i6.682. 36335453

[rda70176-bib-0008] Carballo, G. B. , J. R. Honorato , G. P. F. De Lopes , and T. C. L. D. Spohr . 2018. “A Highlight on Sonic Hedgehog Pathway.” Cell Communication and Signaling 16, no. 1: 1–15. 10.1186/s12964-018-0220-7. 29558958 PMC5861627

[rda70176-bib-0009] Carteau, D. , D. Bassani , and I. Pianet . 2008. “The “Ouzo Effect”: Following the Spontaneous Emulsification of Trans‐Anethole in Water by NMR.” Comptes Rendus Chimie 11, no. 4–5: 493–498. 10.1016/j.crci.2007.11.003.

[rda70176-bib-0010] Chainy, G. B. N. , S. K. Manna , M. M. Chaturvedi , and B. B. Aggarwal . 2000. “Anethole Blocks Both Early and Late Cellular Responses Transduced by Tumor Necrosis Factor: Effect on NF‐κB, AP‐1, JNK, MAPKK and Apoptosis.” Oncogene 19, no. 25: 2943–2950. 10.1038/sj.onc.1203614. 10871845

[rda70176-bib-0011] Chandran, A. , N. J. Merlin , L. Ammu , and S. S. Dharan . 2019. “Fennel Treatment to Pcos: An Insilico Evaluation to Explore the Therapeutic Efficacy of Anethole.” Research Journal of Pharmacy and Technology 12, no. 10: 4958–4962. 10.5958/0974-360X.2019.00860.6.

[rda70176-bib-0012] Cho, H. I. , K. M. Kim , J. H. Kwak , S. K. Lee , and S. M. Lee . 2013. “Protective Mechanism of Anethole on Hepatic Ischemia/Reperfusion Injury in Mice.” Journal of Natural Products 76, no. 9: 1717–1723. 10.1021/np4004323. 23962021

[rda70176-bib-0013] Choo, E. , Y. H. Rhee , S. J. Jeong , et al. 2011. “Anethole Exerts Antimetatstaic Activity via Inhibition of Matrix Metalloproteinase 2/9 and AKT/Mitogen‐Activated Kinase/Nuclear Factor Kappa B Signaling Pathways.” Biological & Pharmaceutical Bulletin 34, no. 1: 41–46. 21212515 10.1248/bpb.34.41

[rda70176-bib-0014] Clark, G. S. 1993. “An Aroma Chemical Profile: Anethole.” Perfumer & Flavorist 18, no. 5: 11–18.

[rda70176-bib-0015] Conceição‐Santos, A. L. , A. C. A. Ferreira , N. A. R. Sá , et al. 2024. “Anethole Supplementation During *in Vitro* Maturation Increases *in Vitro* Goat Embryo Production in a Concentration‐Dependent Manner.” Theriogenology 215: 78–85. 10.1016/j.theriogenology.2023.11.024. 38016304

[rda70176-bib-0016] Conceição‐Santos, A. L. , A. F. B. Silva , N. A. R. Sá , et al. 2025. “Anethole Addition During *in Vitro* Follicle Culture and Oocyte *in Vitro* Maturation Improves Cumulus–Oocyte Complexes Quality and Embryo Production Rates.” Theriogenology 202: 117640. 10.1016/j.theriogenology.2025.117640. 40848425

[rda70176-bib-0017] Conceição‐Santos, A. L. , J. M. D. S. Velarde , D. C. Joaquim , and J. R. Figueiredo . 2025. “The Efficacy of Trans‐Anethole Supplementation During *in Vitro* Culture of Follicles, Oocytes and Embryos: A Meta‐Analysis Approach.” Theriogenology 246: 117557. 10.1016/j.theriogenology.2025.117557. 40577931

[rda70176-bib-0018] Contant, C. , M. Rouabhia , L. Loubaki , F. Chandad , and A. Semlali . 2021. “Anethole Induces Anti‐Oral Cancer Activity by Triggering Apoptosis, Autophagy and Oxidative Stress and by Modulation of Multiple Signaling Pathways.” Scientific Reports 11, no. 1: 13087. 10.1038/s41598-021-92456-w. 34158560 PMC8219795

[rda70176-bib-0019] Domiciano, T. P. , M. M. H. D. O. Dalalio , E. L. Silva , et al. 2013. “Inhibitory Effect of Anethole in Nonimmune Acute Inflammation.” Naunyn‐Schmiedeberg's Archives of Pharmacology 386, no. 4: 331–338. 10.1007/s00210-012-0820-5. 23250338

[rda70176-bib-0020] Duță, D. E. , A. Culețu , M. Negoiță , and V. Ionescu . 2019. “Quantification of Anethole in Fennel and Anise Essential Oils Using Gas Chromatography and 1H‐NMR‐Spectroscopy.” Bulletin of University of Agricultural Sciences and Veterinary Medicine Cluj‐Napoca. Food Science and Technology 76, no. 2: 105–113. 10.15835/buasvmcn-fst:2019.0020.

[rda70176-bib-0021] Feng, T. , L. Zhou , S. Gai , et al. 2019. “ *Acacia catechu* (L.f.) Willd and Scutellaria Baicalensis Georgi Extracts Suppress LPS‐Induced Pro‐Inflammatory Responses Through NF‐кB, MAPK, and PI3K‐Akt Signaling Pathways in Alveolar Epithelial Type II Cells.” Phytotherapy Research 33, no. 12: 3251–3260. 10.1002/ptr.6499. 31506998

[rda70176-bib-0022] Ghasemi‐dehnoo, M. , A. A. Safari , M. Rahimi‐madiseh , Z. Lorigooini , M. T. Moradi , and H. Amini‐khoei . 2022. “Anethole Ameliorates Acetic Acid‐Induced Colitis in Mice: Anti‐Inflammatory and Antioxidant Effects.” Evidence‐Based Complementary and Alternative Medicine 2022: 1–7. 10.1155/2022/9057451. PMC9007635 35432569

[rda70176-bib-0023] Gómez‐Chávez, F. , D. Correa , P. Navarrete‐Meneses , J. C. Cancino‐Diaz , M. E. Cancino‐Diaz , and S. Rodríguez‐Martínez . 2021. “NF‐κB and Its Regulators During Pregnancy.” Frontiers in Immunology 12: 1–12. 10.3389/fimmu.2021.679106. PMC8131829 34025678

[rda70176-bib-0024] Guan, H. Y. , H. X. Xia , X. Y. Chen , L. Wang , Z. J. Tang , and W. Zhang . 2021. “Toll‐Like Receptor 4 Inhibits Estradiol Secretion via NF‐κB Signaling in Human Granulosa Cells.” Frontiers in Endocrinology 12: 1–11. 10.3389/fendo.2021.629554. PMC7995891 33776924

[rda70176-bib-0025] Guo, J. , T. Zhang , Y. Guo , et al. 2018. “Oocyte Stage‐Specific Effects of MTOR Determine Granulosa Cell Fate and Oocyte Quality in Mice.” Proceedings of the National Academy of Sciences of the United States of America 115, no. 23: E5326–E5333. 10.1073/pnas.1800352115. 29784807 PMC6003357

[rda70176-bib-0026] Guo, Z. , and Q. Yu . 2019. “Role of mTOR Signaling in Female Reproduction.” Frontiers in Endocrinology 10. 10.3389/fendo.2019.00692. PMC6794368 31649622

[rda70176-bib-0027] Han, L. , Y. Huang , B. Li , et al. 2023. “The Metallic Compound Promotes Primordial Follicle Activation and Ameliorates Fertility Deficits in Aged Mice.” Theranostics 13, no. 10: 3131–3148. 10.7150/thno.82553. 37351158 PMC10283063

[rda70176-bib-0028] He, Y. , M. M. Sun , G. G. Zhang , et al. 2021. “Targeting PI3K/Akt Signal Transduction for Cancer Therapy.” Signal Transduction and Targeted Therapy 6, no. 1. 10.1038/s41392-021-00828-5. PMC8677728 34916492

[rda70176-bib-0029] Helal, E. G. E. , M. A. Abd‐El‐Aziz , and S. S. Ahmed . 2019. “Effect of Anise (*Pimpinella Anisum* L.) as Phytoestrogen on Some Sex Hormones and Biochemical Parameters.” Egyptian Journal of Hospital Medicine 75, no. 1: 1918–1922. 10.21608/ejhm.2019.29114.

[rda70176-bib-0030] Huang, T. L. , Y. C. Chang , B. C. K. Tsai , et al. 2024. “Anethole Mitigates H_2_O_2_‐Induced Inflammation in HIG‐82 Synoviocytes by Suppressing the Aquaporin 1 Expression and Activating the Protein Kinase A Pathway.” Environmental Toxicology 39, no. 2: 965–978. 10.1002/tox.24023. 37987213

[rda70176-bib-0031] Huang, Y. , J. Zhao , L. Zhou , et al. 2010. “Antifungal Activity of the Essential Oil of *illicium verum* Fruit and Its Main Component Trans‐Anethole.” Molecules 15, no. 11: 7558–7569. 10.3390/molecules15117558. 21030909 PMC6259245

[rda70176-bib-0032] Janini, L. C. Z. , T. T. Dellaqua , C. M. B. Membrive , et al. 2023. “Effects of Anethole Supplementation on Bovine Embryo Production and Quality.” Livestock Science 274: 105262. 10.1016/j.livsci.2023.105262.

[rda70176-bib-0033] Joo, Y. E. , P. S. Jeong , S. Lee , et al. 2023. “Anethole Improves the Developmental Competence of Porcine Embryos by Reducing Oxidative Stress via the Sonic Hedgehog Signaling Pathway.” Journal of Animal Science and Biotechnology 14, no. 1. 10.1186/s40104-022-00824-x. PMC9945695 36814325

[rda70176-bib-0034] Kang, P. , K. Y. Kim , H. S. Lee , S. S. Min , and G. H. Seol . 2013. “Anti‐Inflammatory Effects of Anethole in Lipopolysaccharide‐Induced Acute Lung Injury in Mice.” Life Sciences 93, no. 24: 955–961. 10.1016/j.lfs.2013.10.014. 24404587

[rda70176-bib-0035] Keane, J. A. , and A. D. Ealy . 2024. “An Overview of Reactive Oxygen Species Damage Occurring During *in Vitro* Bovine Oocyte and Embryo Development and the Efficacy of Antioxidant Use to Limit These Adverse Effects.” Animals 14, no. 2. 10.3390/ani14020330. PMC10812430 38275789

[rda70176-bib-0036] Khodadadian, R. , and S. Balali‐ Dehkordi . 2025. “A Comprehensive Review of the Neurological Effects of Anethole.” IBRO Neuroscience Reports 18: 50–56. 10.1016/j.ibneur.2024.12.012. 39844944 PMC11750503

[rda70176-bib-0037] Khoshnam, S. E. , A. Sarkaki , Y. Farbood , A. Keshavarz Zarjani , M. Ghasemi Dehcheshmeh , and S. Moradi Vastegani . 2025. “Anethole Ameliorates Scopolamine‐Induced Memory Deficits and Neuronal Damage Through Antioxidant, Anti‐Inflammatory, and Anticholinesterase Activities in Rats.” Neurochemical Research 50, no. 3. 10.1007/s11064-025-04417-8. 40366448

[rda70176-bib-0038] Kim, K. Y. , H. S. Lee , and G. H. Seol . 2017. “Anti‐Inflammatory Effects of Trans‐Anethole in a Mouse Model of Chronic Obstructive Pulmonary Disease.” Biomedicine & Pharmacotherapy 91: 925–930. 10.1016/j.biopha.2017.05.032. 28511344

[rda70176-bib-0039] Lal, M. , T. Begum , R. Gogoi , et al. 2022. “Anethole Rich Clausena Heptaphylla (Roxb.) Wight & Arn., Essential Oil Pharmacology and Genotoxic Efficiencies.” Scientific Reports 12, no. 1. 10.1038/s41598-022-13511-8. PMC9200763 35705583

[rda70176-bib-0040] Lei, J. D. , S. B. Zhang , W. Z. Ding , et al. 2023. “Antifungal Effects of Trans‐Anethole, the Main Constituent of *Illicium Verum* Fruit Volatiles, on Aspergillus Flavus in Stored Wheat.” Food Control 149, no. March: 109721. 10.1016/j.foodcont.2023.109721.

[rda70176-bib-0041] Liang, C. G. , Y. Q. Su , H. Y. Fan , H. Schatten , and Q. Y. Sun . 2007. “Mechanisms Regulating Oocyte Meiotic Resumption: Roles of Mitogen‐Activated Protein Kinase.” Molecular Endocrinology 21, no. 9: 2037–2055. 10.1210/me.2006-0408. 17536005

[rda70176-bib-0042] Liu, S. , W. Wang , H. Liu , et al. 2024. “Berberine Promotes Primordial Follicle Activation and Increases Ovulated Oocyte Quantity in Aged Mice.” Molecular Medicine 30, no. 1: 251. 10.1186/s10020-024-01042-z. 39707173 PMC11660874

[rda70176-bib-0043] Liu, Y. , X. Zhu , C. Wu , Y. Lang , W. Zhao , and Y. Li . 2022. “Melatonin Protects Against Ovarian Damage by Inhibiting Autophagy in Granulosa Cells in Rats.” Clinics 77: 100119. 10.1016/j.clinsp.2022.100119. 36194922 PMC9531038

[rda70176-bib-0044] Loboda, A. , M. Damulewicz , E. Pyza , A. Jozkowicz , and J. Dulak . 2016. “Role of Nrf2/HO‐1 System in Development, Oxidative Stress Response and Diseases: An Evolutionarily Conserved Mechanism.” Cellular and Molecular Life Sciences 73, no. 17: 3221–3247. 10.1007/s00018-016-2223-0. 27100828 PMC4967105

[rda70176-bib-0045] Lu, C.‐W. , A. Yabuuchi , L. Chen , S. Viswanathan , K. Kim , and G. Q. Daley . 2008. “Ras‐Mitogen Activated Protein Kinase Signaling Promotes Trophectoderm Formation From Embryonic Stem Cells and Murine Embryos.” Nature Genetics 40, no. 7: 921–926. 10.1038/ng.173.Ras-Mitogen. 18536715 PMC2690707

[rda70176-bib-0046] Luo, T. , F. Wang , S. Weng , et al. 2020. “Anethole Compromises Human Sperm Function by Affecting the Sperm Intracellular Calcium Concentration and Tyrosine Phosphorylation.” Reproductive Toxicology 93, no. November 2019: 99–105. 10.1016/j.reprotox.2020.01.007. 32004625

[rda70176-bib-0047] Ma, Q. 2013. “Role of Nrf2 in Oxidative Stress and Toxicity.” Annual Review of Pharmacology and Toxicology 53: 401–426. 10.1146/annurev-pharmtox-011112-140320. PMC4680839 23294312

[rda70176-bib-0048] Marinov, V. , and S. Valcheva‐Kuzmanova . 2015. “Review on the Pharmacological Activities of Anethole.” Scripta Scientifica Pharmaceutica 2, no. 2: 14. 10.14748/ssp.v2i2.1141.

[rda70176-bib-0049] Matboli, M. , A. H. Hasanin , S. Hamady , et al. 2022. “Anti‐Inflammatory Effect of Trans‐Anethol in a Rat Model of Myocardial Ischemia‐Reperfusion Injury.” Biomedicine & Pharmacotherapy 150, no. May: 113070. 10.1016/j.biopha.2022.113070. 35658236

[rda70176-bib-0050] Mohamed, M. E. , M. Kandeel , H. M. A. El‐Lateef , H. S. El‐Beltagi , and N. S. Younis . 2022. “The Protective Effect of Anethole Against Renal Ischemia/Reperfusion: The Role of the TLR2,4/MYD88/NFκB Pathway.” Antioxidants 11, no. 3. 10.3390/antiox11030535. PMC8944622 35326185

[rda70176-bib-0051] Moradi, J. , F. Abbasipour , J. Zaringhalam , et al. 2014. “Anethole, a Medicinal Plant Compound, Decreases the Production of Pro‐Inflammatory TNF‐α and IL‐1β in a Rat Model of LPS‐Induced Periodontitis.” Iranian Journal of Pharmaceutical Research 13, no. 4: 1319–1325. 25587321 PMC4232798

[rda70176-bib-0052] Negahdari, F. M. , M. A. R. Hadjzadeh , Z. Gholamnezhad , Z. S. Noshahr , and Z. Keshavarzi . 2021. “A Comparison Between the Effect of Trans‐Anethole and Metformin on Biochemical Parameters of Polycystic Ovary Syndrome in Rats.” Avicenna Journal of Phytomedicine 11, no. 5: 484–493. 10.22038/AJP.2021.55679.2785. 34745920 PMC8554281

[rda70176-bib-0053] Negahdari, F. M. , M. A. R. Hadjzadeh , Z. Gholamnezhad , F. Sohrabi , and Z. S. Noshahr . 2022. “The Protective Effects of Trans‐Anethole Against Polycystic Ovary Syndrome Induced Histopathological and Metabolic Changes in Rat.” International Journal of Fertility and Sterility 16, no. 3: 192–199. 10.22074/IJFS.2021.532941.1148. 36029056 PMC9395999

[rda70176-bib-0054] Ngo, V. , and M. L. Duennwald . 2022. “Nrf2 and Oxidative Stress: A General Overview of Mechanisms and Implications in Human Disease.” Antioxidants 11, no. 12. 10.3390/antiox11122345. PMC9774434 36552553

[rda70176-bib-0055] Noreen, S. , H. u. Rehman , T. Tufail , H. Badar Ul Ain , and C. G. Awuchi . 2023. “Secoisolariciresinol Diglucoside and Anethole Ameliorate Lipid Abnormalities, Oxidative Injury, Hypercholesterolemia, Heart, and Liver Conditions.” Food Science & Nutrition 11, no. 6: 2620–2630. 10.1002/fsn3.3250. 37324915 PMC10261738

[rda70176-bib-0056] Oliveira, A. C. , J. H. Leal‐Cardoso , C. F. Santos , S. M. Morais , and A. N. Coelho‐de‐Souza . 2001. “Antinociceptive Effects of the Essential Oil of Croton Zehntneri in Mice.” Brazilian Journal of Medical and Biological Research 34, no. 11: 1471–1474. 10.1590/S0100-879X2001001100016. 11668359

[rda70176-bib-0057] O'Rourke, S. A. , L. C. Shanley , and A. Dunne . 2024. “The Nrf2‐HO‐1 System and Inflammaging.” Frontiers in Immunology 15: 1–10. 10.3389/fimmu.2024.1457010. PMC11458407 39380993

[rda70176-bib-0058] Pandit, K. , A. Kumar , S. Kaur , et al. 2022. “Amelioration of Oxidative Stress by Trans‐Anethole via Modulating Phase I and Phase II Enzymes Against Hepatic Damage Induced by CCl4 in Male Wistar Rats.” Environmental Science and Pollution Research 29, no. 4: 6317–6333. 10.1007/s11356-021-16070-z. 34453252

[rda70176-bib-0059] Peerakam, N. , P. Phoowiang , S. Chansakaow , C. Thongpoon , and S. Hadpech . 2022. “Chemical Profiling Revealed a Dominant Compound Trans‐Anethole and Biological Evaluation of an Edible Plant Clausena Harmandiana Containing Essential Oil.” Records of Natural Products 16, no. 2: 118–127. 10.25135/rnp.259.21.03.2025.

[rda70176-bib-0060] Ponte, E. L. , A. F. Pires , D. F. Araujo , et al. 2025. “Inhibitory Effect of Trans‐Anethole in Acute Inflammation: Involvement of Macrophage‐Derived Mediators.” Anais da Academia Brasileira de Ciências 97, no. 1: 1–15. 10.1590/0001-3765202520231288. 40136195

[rda70176-bib-0061] Qu, H. , Y. Zhang , R. He , N. Lin , and C. Wang . 2021. “Anethole Inhibits RANKL‐Induced Osteoclastogenesis by Downregulating ERK/AKT Signaling and Prevents Ovariectomy‐Induced Bone Loss *in Vivo* .” International Immunopharmacology 100: 108113. 10.1016/j.intimp.2021.108113. 34530203

[rda70176-bib-0062] Rakha, S. I. , M. A. Elmetwally , H. El‐Sheikh Ali , A. Balboula , A. M. Mahmoud , and S. M. Zaabel . 2022. “Importance of Antioxidant Supplementation During *in Vitro* Maturation of Mammalian Oocytes.” Veterinary Sciences 9, no. 8. 10.3390/vetsci9080439. PMC9415395 36006354

[rda70176-bib-0063] Raposo, A. , D. Raheem , R. P. Zandonadi , et al. 2024. “Anethole in Cancer Therapy: Mechanisms, Synergistic pHyungseo Bobbyotential, and Clinical Challenges.” Biomedicine & Pharmacotherapy 180. 10.1016/j.biopha.2024.117449. 39326099

[rda70176-bib-0064] Rhee, Y. H. , J. H. Moon , J. H. Mo , T. Pham , and P. S. Chung . 2018. “mTOR and ROS Regulation by Anethole on Adipogenic Differentiation in Human Mesenchymal Stem Cells.” BMC Cell Biology 19, no. 1: 12. 10.1186/s12860-018-0163-2. 29980168 PMC6035441

[rda70176-bib-0065] Ritter, A. M. V. , T. P. Domiciano , W. A. Verri , et al. 2013. “Antihypernociceptive Activity of Anethole in Experimental Inflammatory Pain.” Inflammopharmacology 21, no. 2: 187–197. 10.1007/s10787-012-0152-6. 23054333

[rda70176-bib-0066] Ritter, A. M. V. , L. Hernandes , B. A. da Rocha , et al. 2017. “Anethole Reduces Inflammation and Joint Damage in Rats With Adjuvant‐Induced Arthritis.” Inflammation Research 66, no. 8: 725–737. 10.1007/s00011-017-1053-3. 28547123

[rda70176-bib-0067] Rostami‐Faradonbeh, N. , H. Amini‐Khoei , E. Zarean , E. Bijad , and Z. Lorigooini . 2024. “Anethole as a Promising Antidepressant for Maternal Separation Stress in Mice by Modulating Oxidative Stress and Nitrite Imbalance.” Scientific Reports 14, no. 1: 1–10. 10.1038/s41598-024-57959-2. 38565927 PMC10987547

[rda70176-bib-0068] Ryu, S. , G. H. Seol , H. Park , and I.‐Y. Choi . 2014. “Trans‐Anethole Protects Cortical Neuronal Cells Against Oxygen‐Glucose Deprivation/Reoxygenation.” Neurological Sciences 35, no. 10: 1541–1547. 10.1007/s10072-014-1791-8. 24777545

[rda70176-bib-0069] Sá, N. A. R. , V. R. Araújo , H. H. V. Correia , et al. 2017. “Anethole Improves the *in Vitro* Development of Isolated Caprine Secondary Follicles.” Theriogenology 89: 226–234. 10.1016/j.theriogenology.2015.12.014. 28043356

[rda70176-bib-0070] Sá, N. A. R. R. , J. B. Bruno , D. D. Guerreiro , et al. 2018. “Anethole Reduces Oxidative Stress and Improves *in Vitro* Survival and Activation of Primordial Follicles.” Brazilian Journal of Medical and Biological Research 51, no. 8: e7129. 10.1590/1414-431x20187129. 29846431 PMC5999067

[rda70176-bib-0071] Sá, N. A. R. , A. C. A. Ferreira , F. G. C. Sousa , et al. 2020. “First Pregnancy After *in Vitro* Culture of Early Antral Follicles in Goats: Positive Effects of Anethole on Follicle Development and Steroidogenesis.” Molecular Reproduction and Development 87, no. 9: 966–977. 10.1002/mrd.23410. 32761832

[rda70176-bib-0072] Sá, N. A. R. R. , L. A. Vieira , A. C. A. Ferreira , et al. 2019. “Anethole Supplementation During Oocyte Maturation Improves *in Vitro* Production of Bovine Embryos.” Reproductive Sciences 27, no. 8: 1602–1608. 10.1007/s43032-020-00190-x. 32436196

[rda70176-bib-0073] Salimian, S. , S. Habibian‐Dehkordi , J. Kaboutari , H. Amini‐Khoei , and M. Sajad Barkhordari . 2022. “Anethole Exerted Anticonvulsant Effect in Pentylenetetrazole‐Model of Seizure in Male Mice: Possible Antioxidant Effects.” Future Natural Products 8, no. 1: 2–6. 10.34172/fnp.2022.02.

[rda70176-bib-0074] Samadi‐Noshahr, Z. , M. A. R. Hadjzadeh , R. Moradi‐Marjaneh , and A. Khajavi‐Rad . 2021. “The Hepatoprotective Effects of Fennel Seeds Extract and Trans‐Anethole in Streptozotocin‐Induced Liver Injury in Rats.” Food Science & Nutrition 9, no. 2: 1121–1131. 10.1002/fsn3.2090. 33598196 PMC7866591

[rda70176-bib-0075] Senatore, F. , F. Oliviero , E. Scandolera , et al. 2013. “Chemical Composition, Antimicrobial and Antioxidant Activities of Anethole‐Rich Oil From Leaves of Selected Varieties of Fennel [ *Foeniculum vulgare* Mill. Ssp. Vulgare Var. Azoricum (Mill.) Thell].” Fitoterapia 90: 214–219. 10.1016/j.fitote.2013.07.021. 23933237

[rda70176-bib-0076] Seo, J. W. , S. U. Habiba , Y. A. Munni , et al. 2024. “Protective Effects of Anethole in *Foeniculum Vulgare* Mill. Seed Ethanol Extract on Hypoxia/Reoxygenation Injury in H9C2 Heart Myoblast Cells.” Antioxidants 13, no. 10: 1–18. 10.3390/antiox13101161. PMC11504384 39456415

[rda70176-bib-0077] Silva, F. F. , F. das Chagas Costa , V. A. N. Azevedo , et al. 2024. “ *Croton Grewioides* Essential Oil and Anethole Reduce Oxidative Stress and Improve Growth of Bovine Primordial Follicles During Culture of Ovarian Tissue.” Journal of Pharmacy and Pharmacology 76: 1609–1619. 10.1093/jpp/rgae093. 39016304

[rda70176-bib-0078] Silva, R. , R. Barberino , and M. Matos . 2023. “Impact of Antioxidant Supplementation During *in Vitro* Culture of Ovarian Preantral Follicles: A Review.” Theriogenology 207: 110–122. 10.1016/j.theriogenology.2023.05.027. 37290274

[rda70176-bib-0079] Simcox, J. , and D. W. Lamming . 2022. “The Central moTOR of Metabolism.” Developmental Cell 57, no. 6: 691–706. 10.1016/j.devcel.2022.02.024. 35316619 PMC9004513

[rda70176-bib-0080] Skalicka‐Woźniak, K. , M. Walasek , A. Ludwiczuk , and K. Głowniak . 2013. “Isolation of Terpenoids From *Pimpinella anisum* Essential Oil by High‐Performance Counter‐Current Chromatography.” Journal of Separation Science 36, no. 16: 2611–2614. 10.1002/jssc.201300407. 23749680

[rda70176-bib-0081] Su, Y. Q. , Y. Yin , J. Guo , X. Gong , Y. Tian , and L. Shi . 2022. “MTOR‐Mediated Interaction Between the Oocyte and Granulosa Cells Regulates the Development and Function of Both Compartments in Mice.” Biology of Reproduction 107, no. 1: 76–84. 10.1093/biolre/ioac099. 35552649

[rda70176-bib-0082] Sun, X. , Y. Su , Y. He , et al. 2015. “New Strategy for *in Vitro* Activation of Primordial Follicles With mTOR and PI3K Stimulators.” Cell Cycle 14, no. 5: 721–731. 10.1080/15384101.2014.995496. 25590233 PMC4615062

[rda70176-bib-0083] Tabanca, N. , S. I. Khan , E. Bedir , et al. 2004. “Estrogenic Activity of Isolated Compounds and Essential Oils of Pimpinella Species From Turkey, Evaluated Using a Recombinant Yeast Screen.” Planta Medica 70, no. 8: 728–735. 10.1055/s-2004-827203. 15368661

[rda70176-bib-0084] Tong, Y. , C. Yu , S. Chen , X. Zhang , Z. Yang , and T. Wang . 2023. “Trans‐Anethole Exerts Protective Effects on Lipopolysaccharide‐Induced Acute Jejunal Inflammation of Broilers via Repressing NF‐κB Signaling Pathway.” Poultry Science 102, no. 2: 102397. 10.1016/j.psj.2022.102397. PMC9801195 36565631

[rda70176-bib-0085] Tong, Y. , C. Yu , Z. Xie , X. Zhang , Z. Yang , and T. Wang . 2022. “Trans‐Anethole Ameliorates Lipopolysaccharide‐Induced Acute Liver Inflammation in Broilers via Inhibiting NF‐κB Signaling Pathway.” Poultry Science 101, no. 8: 101962. 10.1016/j.psj.2022.101962. PMC9192971 35690001

[rda70176-bib-0086] Vastegani, S. M. , S. E. Khoshnam , E. Mansouri , et al. 2023. “Neuroprotective Effect of Anethole Against Rotenone Induced Non‐Motor Deficits and Oxidative Stress in Rat Model of Parkinson's Disease.” Behavioural Brain Research 437, no. July 2022: 114100. 10.1016/j.bbr.2022.114100. 36075399

[rda70176-bib-0087] Wang, X. , C. Li , Y. Wang , L. Li , Z. Han , and G. Wang . 2020. “UFL1 Alleviates LPS‐Induced Apoptosis by Regulating the NF‐κB Signaling Pathway in Bovine Ovarian Granulosa Cells.” Biomolecules 10, no. 2: 260. 10.3390/biom10020260. 32050508 PMC7072671

[rda70176-bib-0088] Waza, A. A. , Z. Hamid , S. Ali , S. A. Bhat , and M. A. Bhat . 2018. “A Review on Heme Oxygenase‐1 Induction: Is It a Necessary Evil.” Inflammation Research 67, no. 7: 579–588. 10.1007/s00011-018-1151-x. 29693710

[rda70176-bib-0089] Wright, C. J. , E. L. Cari , J. Sandoval , et al. 2020. “Control of Murine Primordial Follicle Growth Activation by IκB/NFκB Signaling.” Reproductive Sciences 27, no. 11: 2063–2074. 10.1007/s43032-020-00225-3. 32542534 PMC7529825

[rda70176-bib-0090] Xu, H. , F. Chen , Z. Liu , et al. 2024. “B(a)P Induces Ovarian Granulosa Cell Apoptosis via TRAF2‐NFκB‐Caspase1 Axis During Early Pregnancy.” Environmental Research 252: 118865. 10.1016/j.envres.2024.118865. 38583661

[rda70176-bib-0091] Yadollahi‐Farsani, Y. , V. R. Vanani , Z. Lorigooini , A. Farahzad , and H. Amini‐Khoei . 2024. “Anethole via Increase in the Gene Expression of PI3K/AKT/mTOR Mitigates the Autistic‐Like Behaviors Induced by Maternal Separation Stress in Mice.” IBRO Neuroscience Reports 16, no. November 2023: 1–7. 10.1016/j.ibneur.2023.11.009. 38145174 PMC10733685

[rda70176-bib-0092] Yancu, D. , C. Vaillancourt , and J. T. Sanderson . 2019. “Evaluating the Effects on Steroidogenesis of Estragole and Trans‐Anethole in a Feto‐Placental Co‐Culture Model.” Molecular and Cellular Endocrinology 498: 110583. 10.1016/j.mce.2019.110583. 31536780

[rda70176-bib-0093] Yang, E. C. , Y. Y. Hsieh , and L. Y. Chuang . 2021. “Comparison of the Phytochemical Composition and Antibacterial Activities of the Various Extracts From Leaves and Twigs of *illicium verum* .” Molecules 26, no. 13. 10.3390/molecules26133909. PMC8272203 34206777

[rda70176-bib-0094] Younis, N. S. , and M. E. Mohamed . 2022. “Anethole's Effects Against Myocardial Infarction: The Role of TLR4/NFκB and Nrf2/HO1 Pathways.” Chemico‐Biological Interactions 360, no. August 2021: 109947. 10.1016/j.cbi.2022.109947. 35430261

[rda70176-bib-0095] Younis, N. S. , and M. E. Mohamed . 2023. “Anethole Pretreatment Modulates Cerebral Ischemia/Reperfusion: The Role of JNK, p38, MMP‐2 and MMP‐9 Pathways.” Pharmaceuticals (Basel) 16, no. 3. 10.3390/ph16030442. PMC10057436 36986541

[rda70176-bib-0096] Yu, C. , Y. Tong , Q. Li , T. Wang , and Z. Yang . 2022. “Trans‐Anethole Ameliorates Intestinal Injury Through Activation of Nrf2 Signaling Pathway in Subclinical Necrotic Enteritis‐Induced Broilers.” Frontiers in Veterinary Science 9. 10.3389/fvets.2022.877066. PMC9062583 35518639

[rda70176-bib-0097] Yu, C. , D. Wang , Q. Li , Y. Tong , Z. Yang , and T. Wang . 2022. “Trans‐Anethole Ameliorates LPS‐Induced Inflammation via Suppression of TLR4/NF‐κB Pathway in IEC‐6 Cells.” International Immunopharmacology 108: 108872. 10.1016/j.intimp.2022.108872. 35617845

[rda70176-bib-0098] Yu, C. , T. Wang , and Z. Yang . 2022. “Effects of Dietary Supplementation of Trans‐Anethole on the Intestinal Antioxidant Status, Immune Function, and Liver Lipid Metabolism in Broilers.” Italian Journal of Animal Science 21, no. 1: 729–736. 10.1080/1828051X.2022.2059021.

[rda70176-bib-0099] Zeni, V. , R. Ricciardi , A. Masoni , et al. 2025. “ *Pimpinella Anisum* Essential Oil and Trans‐Anethole Activity Against Key Insect Pests and Non‐Target Ants.” Journal of Pest Science 98, no. 2: 705–716. 10.1007/s10340-024-01842-6.

[rda70176-bib-0100] Zhang, X. , W. Zhang , Z. Wang , et al. 2022. “Enhanced Glycolysis in Granulosa Cells Promotes the Activation of Primordial Follicles Through mTOR Signaling.” Cell Death & Disease 13, no. 1. 10.1038/s41419-022-04541-1. PMC8795455 35087042

